# Author Correction: Involvement of autophagy in hypoxia-BNIP3 signaling to promote epidermal keratinocyte migration

**DOI:** 10.1038/s41419-019-1533-1

**Published:** 2019-04-01

**Authors:** Junhui Zhang, Can Zhang, Xupin Jiang, Lingfei Li, Dongxia Zhang, Di Tang, Tiantian Yan, Qiong Zhang, Hongping Yuan, Jiezhi Jia, Jiongyu Hu, Jiaping Zhang, Yuesheng Huang

**Affiliations:** 1 Institute of Burn Research, State Key Laboratory of Trauma, Burns and Combined Injury, Southwest Hospital, Army Medical University (Third Military Medical University), Chongqing, China; 2 Military Burn Center, the 990th (159th) Hospital of People’s Liberation Army, Zhumadian, China; 3 Endocrinology Department, Southwest Hospital, Army Medical University (Third Military Medical University), Chongqing, China; 4 Department of Plastic Surgery, Southwest Hospital, Army Medical University (Third Military Medical University), Chongqing, China

**Correction to:** **Cell Death and Disease**

10.1038/s41419-019-1473-9

published online 8 March 2019

Following publication of the original article, it has come to our attention that the Materials and Methods section of the paper was missing. This is because this section was accidentally omitted from the final version of the manuscript when it was submitted to production. Both the PDF and HTML versions of the article have been updated with the missing section and references. As a result, the references at the end of article have been renumbered as well. We apologize for this inconvenience.

